# A tapetal-like fundus reflex in a healthy male: evidence against a role in the pathophysiology of retinal degeneration?

**Published:** 2012-05-02

**Authors:** Patrik Schatz, Jesper Bregnhøj, Henrik Arvidsson, Dror Sharon, Liliana Mizrahi-Meissonnier, Birgit Sander, Karen Grønskov, Michael Larsen

**Affiliations:** 1 Department of Ophthalmology, Glostrup Hospital, University of Copenhagen, Denmark; 2 Department of Ophthalmology, Lund University Hospital, University of Lund, Sweden; 3 National Eye Clinic for the Visually Impaired and Kennedy Center, Glostrup, Denmark; 4 Department of Ophthalmology, Hadassah-Hebrew University Medical Center, Jerusalem, Israel

## Abstract

**Purpose:**

To report on the retinal function and structure in a 37-year-old male who presented with a tapetal-like reflex (TLR) indistinguishable from that seen in female carriers of X-linked retinitis pigmentosa (XLRP).

**Methods:**

Clinical examination included dark adaptometry, full-field electroretinography (ERG), multifocal ERG, optical coherence tomography, and fundus autofluorescence photography. Molecular genetic testing included screening for known mutations in autosomal dominant, autosomal recessive, and X linked retinitis pigmentosa (RP) genes with a commercially available chip, and sequencing analysis of retinitis pigmentosa GTPase regulator (RPGR)-open reading frame 15 (ORF15).

**Results:**

Fundus examination revealed a bilateral TLR, which is typical of female carriers of XLRP. Imaging studies and electrophysiological testing was unremarkable, except for a significant increase in full-field ERG amplitudes after prolonged dark adaptation as compared to after standard dark adaptation. Mutation screening was negative.

**Conclusions:**

TLR was found for the first time, to the best of our knowledge, in a male subject. There were no definitive signs of retinal degeneration, suggesting that this reflex in itself is not necessarily a precursor of the retinal degeneration that can be seen in female carriers of XLRP.

## Introduction

Although their pathophysiology is largely unknown, unusual fundus reflexes may have differential diagnostic implications in retinal degenerations. In Oguchi disease, one of the various congenital stationary night blindnesses, a golden tapetal reflex has been described, which disappears after 2–3 h of dark adaptation; this is referred to as the Mizuo phenomenon [1-3]. The concentration of potassium in the retina has been implicated in the pathogenesis of this phenomenon, since it has been observed in animal studies that a small potassium chloride leak from a defective microelectrode into the inner retina produces a similar yellow-golden sheen [4]. A similar phenomenon has been described in X-linked retinoschisis (XLRS), possibly as a result of a decreased potassium scavenging capacity of retinal Müller cells. The latter assumption could potentially also explain the reduced electroretinographic b-wave in this condition, as it is generated by K^+^ flow through the Müller cells, mainly as a result of light-evoked depolarization of “ON” bipolar neurons [5-7]. Light falling on the retina results in hyperpolarization of the photoreceptors and in increased extracellular potassium. In the dark-adapted state, the extracellular K^+^ concentration is much lower again, with normalization of the reflex. Further conditions associated with abnormal fundus reflectivity include early X-linked retinitis pigmentosa (XLRP) in young males [8] and Sheen retinal dystrophy [9].

The tapetal-like fundus reflex (TLR) of female carriers of XLRP is considered to be specific to them. It is described as brightly scintillating particulate reflection on ophthalmoscopic examination, with relative sparing of the fovea [10]. The origin of this reflex has been subject to image analysis and fundus reflectometry studies [10,11]. It has been suggested that an increase in reflectance of the outer segments of the photoreceptors underlies the TLR [11].

Carriers of XLRP may also display a range of abnormalities of retinal function [12,13]. However, the nature of the relation between the retinal degeneration and the TLR has not been fully determined.

In the following, we report the finding of such a reflex in a healthy 37-year-old male in whom no association with RP could be demonstrated. For comparison, we also include findings from two female carriers of XLRP.

## Methods

The study was approved by an institutional ethics committee and informed consent was obtained. The male patient was examined with full-field electroretinography (ffERG), Goldmann-Weeker dark adaptometry, multifocal electroretinography (mfERG, VERIS 4; EDI, San Mateo, CA), optical coherence tomography (OCT-4; Zeiss Humphrey Instruments, Dublin, CA), and fundus autofluorescence photography (Spectralis HRA-OCT; Heidelberg Engineering, Heidelberg, Germany) was performed as described previously [14,15]. Dark adapted FfERG was performed according to International Society for Clinical Electrophysiology of Vision (ISCEV) standard, as well as after prolonged dark adaptation for 24 h, achieved by overnight patching of the right eye. After topical anesthesia of the eye, a Burian Allen bipolar contact lens (Hansen Labs, Coralville, IA) was applied on the cornea, and a ground electrode was applied on the forehead. Responses were obtained with a wide-band filter (−3 dB at 1 Hz and 500 Hz) stimulated with a single full-field flash (30 µsec) with blue light (Wratten filter 47, 47A, and 47B, Kodak, Rochester, NY) and with white light (3.93 cd/m^2^). Cone responses were obtained with 30-Hz flickering white light (0.81 cd/m^2^) averaged from 20 sweeps.

For comparison, we retrospectively reviewed fundus photography from carriers of XLRP in the photographic archives of our clinic, in search for TLR. In addition, a 33-year-old female carrier of XLRP with no TLR, with a known mutation c.854G>A in retinitis pigmentosa GTPase regulator (*RPGR*) was examined by mfERG and fundus autofluorescence photography as follows. We overlaid the focal ERGs from the mfERG on the fundus autofluorescence photograph, attempting at an exact correspondence between the focal ERGs and their retinal location of origin. For this, we used the projection of the mfERG stimulus hexagons on the infrared fundus image that is present during the mfERG investigation. The mfERG array was then transferred over to the fundus autofluorescence photograph, with an attempt to preserve the same correspondence relative to retinal location.

Molecular genetic testing in the male patient included screening for known autosomal recessive, autosomal dominant, and X-linked RP mutations by a commercially available microarray technique (Asper Ophthalmics, Tartu, Estonia). Previously published mutations in the following genes were screened:

1. The autosomal recessive retinitis pigmentosa (AR-RP) test, including the ceramide kinase-like (*CERKL*), cyclic nucleotide-gated channel alpha 1 (*CNGA1*), cyclic nucleotide gated channel beta 1 *(CNGB1*), C-mer proto-oncogene tyrosine kinase (*MERTK*), phosphodiesterase 6A (*PDE6A*), phosphodiesterase 6B (*PDE6B*), nuclear receptor subfamily 2 group E,member 3 (*NR2E3*), retinol dehydrogenase 12 (*RDH12*), retinal G protein coupled receptor (*RGR*), retinaldehyde binding protein 1 (*RLBP1*), S-antigen (*SAG*), tubby like protein 1 (*TULP1*), Crumbs homolog 1 (*CRB*), retinal pigment epithelium-specific protein 65 kDa (*RPE65*), Usher syndrome 2A (*USH2A*), clarin 1 (*USH3A*), and lecithin retinol acyltransferase (*LRAT*) genes;
2. The AD-RP test, including the carbonic anhydrase IV (*CA4*), Fascin homolog 2 (*FSCN2*), inosine monophosphate dehydrogenase 1 (*IMPDH1*), neural retina leucine zipper (*NRL*), pre-mRNA processing factor 3 homolog (*PRPF3*), pre-mRNA processing factor 31 homolog (*PRPF31*), pre-mRNA processing factor 8 homolog (*PRPF8*), retinal degeneration slow (*RDS*), rhodopsin (*RHO*), retinal outer segment membrane protein 1 (*ROM1*), retinitis pigmentosa 1 (*RP1*), retinitis pigmentosa 9 (*RP9*) and cone-rod homeobox (*CRX*) genes; and
3. The X-linked retinitis pigmentosa (XL-RP) test, including the retinitis pigmentosa 2 (*RP2*) and retinitis pigmentosa GTPase regulator (*RPGR*; only exons 1–14) genes.

As the *RPGR* open reading frame (ORF) 15 is not included in the latter test, we performed direct sequencing of PCR products to screen *RPGR* ORF15 [16].

In addition, PCR analysis of polymorphic microsatellite markers on the X and Y chromosomes (QSTR-XYv2 kit from Elucigene; Gen Probe, San Diego, CA) was performed to determine whether there was a normal male set of chromosomes.

## Results

**Figure 1:**
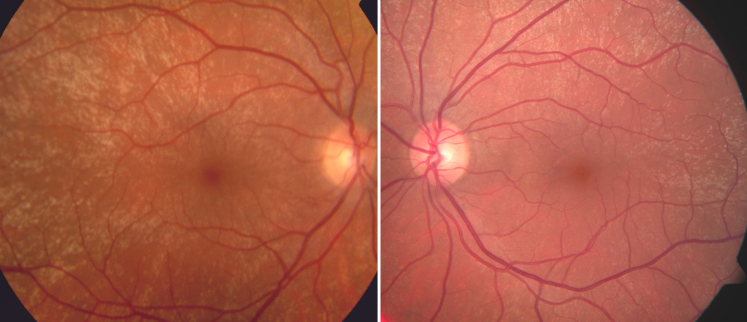
Fundus photography in a healthy male patient demonstrating the presence of bilateral tapetal-like reflex.

A now 37-year-old patient presented at our department more than 10 years before after a trauma to the head region. In a routine examination, we discovered unusual yellowish reflexes scattered in both fundi, extending from the perimacular region into the midperiphery (Figure 1). Peripheral retina was unremarkable, specifically there was no retinoschisis. The patient had two healthy brothers and a healthy daughter. All family members, including mother and father, had normal fundi and fundus photographs (except for presumably age-related scattered drusen around the vascular arcades and in the posterior pole in the mother), full vision, and no visual symptoms. There was no family history of any ophthalmic or other disease, except a reported cataract in a grandparent. The patient was asymptomatic and had a best corrected visual acuity of 1.0 (decimal acuity scale) in both eyes. FfERG was performed to rule out a retinal degeneration, and was found to be normal.

The patient was re-examined 10 years later, including clinical and electrophysiological evaluation and molecular genetic tests.

**Figure 2:**
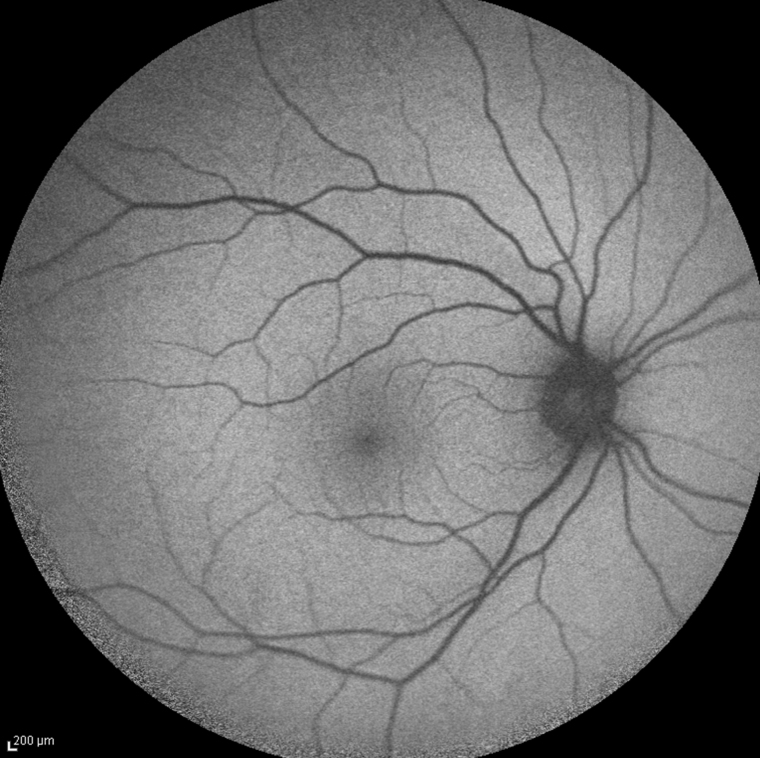
Fundus autofluorescence in the male patient with a tapetal-like reflex, demonstrating no obvious abnormality.

**Figure 3:**
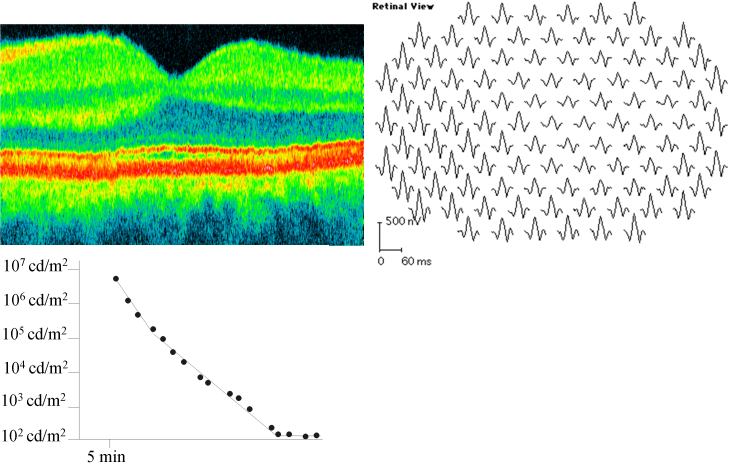
Dark adaptometry (lower left panel), optical coherence tomography (upper left panel), and multifocal electroretinography (upper right panel) in the male patient with a tapetal-like reflex, demonstrating no obvious abnormality of retinal function or structure. The following abbreviations apply: cd is short for candela, m^2^ is short for square meter, ms is short for millisecond and nV is short for nanovolt.

**Figure 4:**
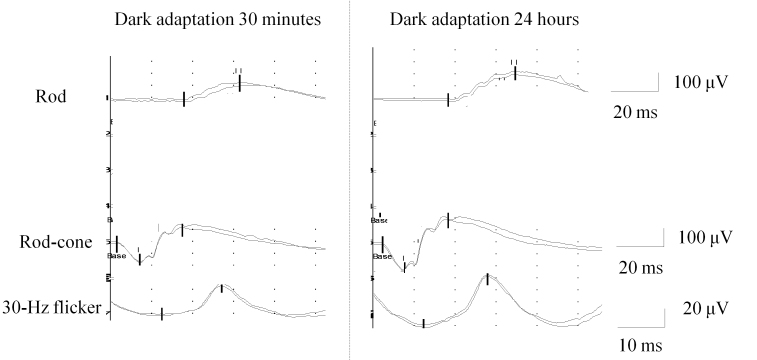
Full-field electroretinography in our male patient with a tapetal-like reflex. Amplitudes and implicit times were within normal limits after standard dark adaptation (left panel); however, a significant increase in response was seen after prolonged dark adaptation (right panel). The following abbreviations apply: μV is short for microvolt and ms is short for milliseconds.

Fundus autofluorescence (Figure 2), dark adaptometry (Figure 3, lower left panel), optical coherence tomography (OCT; Figure 3, upper left panel), and mfERG (Figure 3, upper right panel) were normal. FfERG was within normal limits after the standard 30 min dark adaptation; however, rod and cone responses increased by >50% each after prolonged 24 h dark adaptation (Figure 4, Table 1).

**Table 1:** Full-field electroretinography results.

**Subject**	**Rod amplitude (μV) DA 30 min**	**Rod amplitude (μV) DA 24 h**	**Rod-cone amplitude (μV) DA 30 min**	**Rod-cone amplitude (μV) DA 24 h**	**30 Hz flicker amplitude (μV) DA 30 min**	**30 Hz flicker amplitude (μV) DA 24 h**	**30 Hz flicker implicit time (ms) DA 30 min**	**30 Hz flicker implicit time (ms) DA 24 h**
Male patient	147	234	292	431	55	86	26.9	27.7
**Normal**								
Median	137		305		53		29.1	
Range	64–221		158–546		20–117		25.2–33.2	

All amplitudes in the table refer to b-wave amplitudes. DA: Dark adaptation; μV: microvolt; ms: milliseconds; min: minutes; h: hours; Hz: Herz.

Analysis for known mutations in RP genes revealed no mutations. In addition, a PCR analysis for polymorphic microsatellite markers on the X and Y chromosomes displayed the presence of one X chromosome and one Y chromosome.

**Figure 5:**
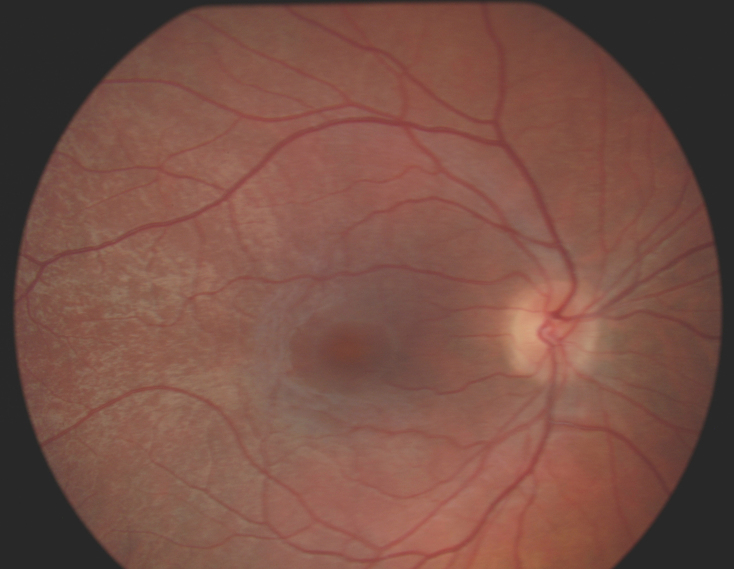
Fundus photography demonstrating a tapetal-like reflex in a 22-year-old female carrier of X-linked retinitis pigmentosa, from the photographic archives of our clinic. The patient belongs to a known Danish X-linked retinitis pigmentosa (XLRP) family, where molecular genetic analysis has identified a disease causing single base pair deletion c.3395delA at the 3′ end of the ORF15 exon in *RPGR*, resulting in a premature stop codon.

In a retrospective review of fundus photography in the photographic archives of our clinic we identified a bilateral TLR in a 22-year old female carrier of XLRP, belonging to a family with the single base pair deletion c.3395delA (g.ORF15+1642delA) at the 3′ end of the ORF15 exon in *RPGR* resulting in a premature stop codon (Figure 5) [17]. Further investigation, for example electrophysiology, was not possible at this point.

**Figure 6:**
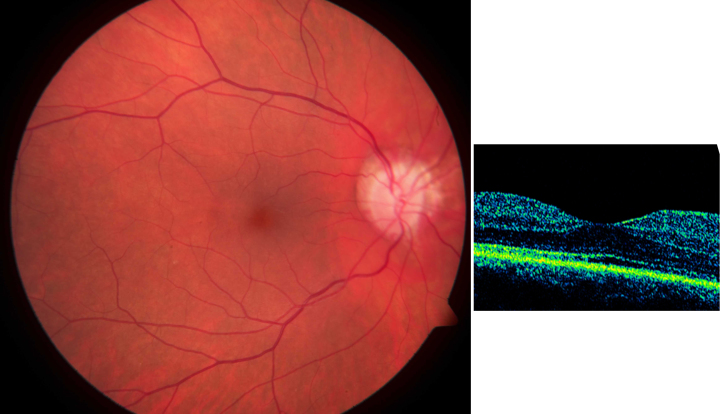
Fundus photography (left panel) and optical coherence tomography (right panel) in a 33-year-old female carrier of X-linked retinitis pigmentosa with a known c.854G>A mutation in *RPGR*, demonstrating no obvious retinal structural abnormalities and no tapetal-like reflex.

**Figure 7:**
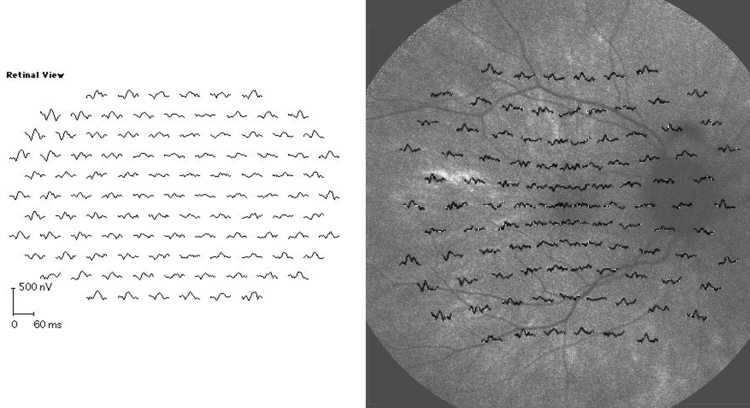
Multifocal electroretinography (left panel) and overlay of multifocal electroretinography focal responses on fundus autofluorescence photography (right panel) in the 33-year-old female carrier of X-linked retinitis pigmentosa whose fundus is presented in Figure 6. Patchy retinal dysfunction compatible with random X inactivation, and a patchy variability in fundus autofluorescence intensity is demonstrated. Areas of remaining autofluorescence seem to correspond to areas of remaining responses by multifocal electroretinography. The following abbreviations apply: ms is short for millisecond and nV is short for nanovolt.

A 33-year-old female carrier with c.854G>A, a known mutation in *RPGR* [18], but in whom no TLR was present, was investigated. The fundus and OCT (Figure 6) did not demonstrate any obvious abnormalities; however, autofluorescence photography and mfERG (Figure 7) demonstrated localized variation of autofluorescence intensity along with areas of focally reduced function by mfERG (Figure 7). MfERG ring averages and OCT foveal thickness from the latter patient and from the male patient with the TLR are presented in Table 2.

**Table 2:** Optical coherence tomography and multifocal electroretinography findings.

**Subject**	**MfERG Ring 1–2 amplitude (nV/deg2)**	**MfERG Ring 1–2 latency(ms)**	**MfERG Ring 3–6 amplitude**	**MfERG Ring 3–6 latency (ms)**	**OCT foveal thickness (μm)**
XLRP carrier	11.4	30	7.4	30.8	199
Male patient	38.9	29.2	27.2	28.3	215
**Normal**					
Median	29.3	26.3	12.7	26.3	182
Range	22.8–35.2	25.8–29.5	10.6–20.2	25.0–28.3	157–207

OCT: Optical coherence tomography. MfERG: multifocal electroretinography. XLRP carrier: 33 year old female carrier of X linked retinitis pigmentosa. ms: milliseconds.

## Discussion

The described male patient presented with bilateral TLR, indistinguishable from that associated with female carriers of XLRP. We confirmed male sex by PCR analysis of the X and Y chromosomes. This also excludes Klinefelter syndrome (47, XXY), which could theoretically explain a phenotype associated with the female sex in a male patient [19]. The TLR of female carriers of XLRP is considered to be specific for this condition, and has not, to the best of our knowledge, been previously described in a male.

Abnormal fundus reflections in male patients may be seen in Oguchi disease, XLRS, sheen retinal dystrophy, and early XLRP [1-9]. However, these conditions are frequently associated with profound retinal degeneration, specific alterations in the ffERG after standard dark adaptation for 30 min and/or structural alterations in the inner retina, none of which was present in our patient. Furthermore, these abnormal reflections differ in appearance from the TLR seen in female carriers of XLRP and in our patient.

Standardized electrophysiological examination including ffERG after 30 min dark adaptation failed to reveal any significant abnormality in our patient. There was no change of fundus appearance over a follow-up period of more than 10 years. The stability of the TLR is also reported for female carriers of XLRS, thus challenging the assumption that the TLR is a stage of the retinal degeneration [10,11]. Our findings related to this reflex in a healthy male seem to support this notion. On the other hand, female carriers usually only present with degeneration later in life. They may have normal ffERG and visual fields, especially in younger age groups [12]. We cannot exclude the possibility that this male patient will develop signs of retinal degeneration later on.

There was a significant and similar (>50%) increase in ffERG rod and cone response amplitudes after prolonged dark adaptation compared to after standard dark adaptation. A similar or even larger magnitude of improvement of responses in the ffERG may be observed in specific disorders due to mutations in visual cycle genes, leading to deficient recycling of 11-cis retinal to reconstitute rhodopsin, for example fundus albipunctatus [15]. However, the latter condition features severely reduced or even nonrecordable responses after standard dark adaptation, in contrast to our patient who presented with ffERG responses within normal limits after standard 30 min dark adaptation. In fundus albipunctatus, white dots visible on fundus exam coincide with focal hyperreflective accumulations that span from Bruch’s membrane across the retinal pigment epithelium and photoreceptor outer and inner segments on OCT [15]. However, our patient presented with normal OCT. Thus, the structural substrate, if any, of the TLR in our subject remains elusive. One reviewer suggested the possibility of a specific distribution of amelanotic retinal pigment epithelium cells, underlying the TLR.

Genetic screening regarding XLRP genes included the two major XLRP genes: *RP2*, which accounts for 10%–20% of recessive XLRP, and *RPGR*, which accounts for 50%–80% of recessive XLRP [20]. However, at least four additional loci are mapped for which the genes are not yet identified [21]. In a recent study, it was suggested that the genetic defects in *RPGR* and *RP2*, which are considered to lead to dysfunction of the connecting cilium of the photoreceptor cells, could result in accumulation of retinoid compounds in the inner segments of the photoreceptors that could account for patches of increased autofluorescence observed in that study [13]. In the present study, we did not notice any abnormalities regarding fundus autofluorescence (Figure 2).

Although well documented in the literature, and considered specific for the condition, we are aware of only three studies that include photographic presentation of the TLR: Cideciyan and Jacobson [10], Wegscheider et al. [22], and recently Genead et al. [13].

For comparison, we present a fundus photograph (Figure 5) of the TLR in a female carrier of XLRP, and we investigated a 33-year-old female carrier of XLRP, without any TLR. (Figure 6 and Figure 7). The patchy reduction of mfERG is compatible with random X inactivation and the Lyon hypothesis [23], and has been demonstrated before in female carriers of XLRP [12]. Interestingly, remaining responses in the mfERG seem to correspond to remaining autofluorescence (Figure 7). It may be suggested that localized photoreceptor apoptosis lead to secondary atrophy of the retinal pigment epithelium, explaining the regional variation of autofluorescence. However, the relation between regional variation in fundus autofluorescence and retinal function may be more complex and requires further investigation.

To conclude, this is to our knowledge the first description of the TLR, which was previously considered specific to female carriers of XLRP, in a healthy male patient, in whom clinical investigations and molecular genetic analysis failed to reveal any definitive association with retinal degeneration. This indicates that, at least until 30–40 years of age or longer, TLR may be present in spite of the absence of further typical signs of retinal degeneration. However, the nature and origin of the phenomenon are still not clear and further studies are required.

## References

[1] Usui T, Ichibe M, Ueki S, Takagi M, Hasegawa S, Abe H, Sekiya K, Nakazawa M (2000). Mizuo phenomenon observed by scanning laser ophthalmoscopy in a patient with Oguchi disease.. Am J Ophthalmol.

[2] Fuchs S, Nakazawa M, Maw M, Tamai M, Oguchi Y, Gal A (1995). A homozygous 1-base pair deletion in the arrestin gene is a frequent cause of Oguchi disease in Japanese.. Nat Genet.

[3] Noble KG, Margolis S, Carr RE (1989). The golden tapetal sheen reflex in retinal disease.. Am J Ophthalmol.

[4] de Jong PT, Zrenner E, van Meel GJ, Keunen JE, van Norren D (1991). Mizuo phenomenon in X-linked retinoschisis. Pathogenesis of the Mizuo phenomenon.. Arch Ophthalmol.

[5] Vincent A, Shetty R, Yadav NK, Shetty BK (2009). Foveal schisis with Mizuo phenomenon: etio-pathogenesis of tapetal reflex in X-linked retinoschisis.. Eye (Lond).

[6] Robson AG, Mengher LS, Tan MH, Moore AT (2009). An unusual fundus phenotype of inner retinal sheen in X-linked retinoschisis.. Eye (Lond).

[7] Miyake Y, Terasaki H (1999). Golden tapetal-like fundus reflex and posterior hyaloid in a patient with x–linked juvenile retinoschisis.. Retina.

[8] Goodman G, Ripps H, Siegel IM (1965). Sex-linked ocular disorders: Trait expressivity in males and carrier females.. Arch Ophthalmol.

[9] Polk TD, Gass JD, Green WR, Novak MA, Johnson MW (1997). Familial internal limiting membrane dystrophy. A new sheen retinal dystrophy.. Arch Ophthalmol.

[10] Cideciyan AV, Jacobson SG (1994). Image analysis of the tapetal-like reflex in carriers of X-linked retinitis pigmentosa.. Invest Ophthalmol Vis Sci.

[11] Berendschot TT, DeLint PJ, van Norren D (1996). Origin of tapetal-like reflexes in carriers of X-linked retinitis pigmentosa.. Invest Ophthalmol Vis Sci.

[12] Vajaranant TS, Seiple W, Szlyk JP, Fishman GA (2002). Detection using the multifocal electroretinogram of mosaic retinal dysfunction in carriers of X-linked retinitis pigmentosa.. Ophthalmology.

[13] Genead MA, Fishman GA, Lindeman M (2010). Structural and functional characteristics in carriers of X-linked retinitis pigmentosa with a tapetal-like reflex.. Retina.

[14] Schatz P, Ponjavic V, Andréasson S, McGee TL, Dryja TP, Abrahamson M (2005). Clinical phenotype in a Swedish family with a mutation in the IMPDH1 gene.. Ophthalmic Genet.

[15] Schatz P, Preising M, Lorenz B, Sander B, Larsen M, Rosenberg T (2011). Fundus Albipunctatus Associated with Compound Heterozygous Mutations in RPE65.. Ophthalmology.

[16] Sharon D, Sandberg MA, Rabe VW, Stillberger M, Dryja TP, Berson EL (2003). RP2 and RPGR mutations and clinicalcorrelations in patients with X-linked retinitis pigmentosa.. Am J Hum Genet.

[17] Neidhardt J, Glaus E, Lorenz B, Netzer C, Li Y, Schambeck M, Wittmer M, Feil S, Kirschner-Schwabe R, Rosenberg T, Cremers FP, Bergen AA, Barthelmes D, Baraki H, Schmid F, Tanner G, Fleischhauer J, Orth U, Becker C, Wegscheider E, Nürnberg G, Nürnberg P, Bolz HJ, Gal A, Berger W (2008). Identification of novel mutations in X-linked retinitis pigmentosa families and implications for diagnostic testing.. Mol Vis.

[18] Roepman R, van Duijnhoven G, Rosenberg T, Pinckers AJ, Bleeker-Wagemakers LM, Bergen AA, Post J, Beck A, Reinhardt R, Ropers HH, Cremers FP, Berger W (1996). Positional cloning of the gene for X-linked retinitis pigmentosa 3: homology with the guanine-nucleotide-exchange factor RCC1.. Hum Mol Genet.

[19] Paduch DA, Bolyakov A, Cohen P, Travis A (2009). Reproduction in men with Klinefelter syndrome: the past, the present, and the future.. Semin Reprod Med.

[20] Veltel S, Wittinghofer A (2009). RPGR and RP2: targets for the treatment of X-linked retinitis pigmentosa?. Expert Opin Ther Targets.

[21] Beltran WA, Acland GM, Aguirre GD (2009). Age-dependent disease expression determines remodeling of the retinal mosaic in carriers of RPGR exon ORF15 mutations.. Invest Ophthalmol Vis Sci.

[22] Wegscheider E, Preising MN, Lorenz B (2004). Fundus autofluorescence in carriers of X-linked recessive retinitis pigmentosa associated with mutations in RPGR, and correlation with electrophysiological and psychophysical data.. Graefes Arch Clin Exp Ophthalmol.

[23] Huynh KD, Lee JT (2005). X-chromosome inactivation: a hypothesis linking ontogeny and phylogeny.. Nat Rev Genet.

